# Impacts of body weight change on treatment outcomes in patients with multidrug-resistant tuberculosis in Northwest Ethiopia

**DOI:** 10.1038/s41598-023-51026-y

**Published:** 2024-01-04

**Authors:** Fasil Wagnew, Kefyalew Addis Alene, Matthew Kelly, Darren Gray

**Affiliations:** 1 College of Health Sciences, Debre Markos University, Debre Markos, Ethiopia; 2 National Centre for Epidemiology and Population Health (NCEPH), College of Health and Medicine, The Australian National University, Canberra, Australia; 3 Geospatial and Tuberculosis Research Team, Telethon Kids Institute, Nedlands, WA Australia; 4 School of Population Health, Faculty of Health Sciences, Curtin University, Bentley, WA Australia; 5 Population Health Program, QIMR Berghofer Medical Research Institute, Brisbane, QLD Australia

**Keywords:** Medical research, Epidemiology, Outcomes research

## Abstract

Measuring body weight during therapy has received insufficient attention in poor resource settings like Ethiopia. We aimed to investigate the association between weight change during therapy and treatment outcomes among patients with multidrug-resistant tuberculosis (MDR-TB) in northwest Ethiopia. This retrospective cohort study analysed data from patients with MDR-TB admitted between May 2015 to February 2022 at four treatment facilities in Northwest Ethiopia. We used the joint model (JM) to determine the association between weight change during therapy and treatment outcomes for patients with MDR-TB. A total of 419 patients with MDR-TB were included in the analysis. Of these, 265 (63.3%) were male, and 255 (60.9%) were undernourished. Weight increase over time was associated with a decrease in unsuccessful treatment outcomes (adjusted hazard ratio (AHR): 0.96, 95% CI: 0.94 to 0.98). In addition, patients with undernutrition (AHR: 1.72, 95% CI: 1.10 to 2.97), HIV (AHR:1.79, 95% CI: 1.04 to 3.06), and clinical complications such as pneumothorax (AHR: 1.66, 95% CI: 1.03 to 2.67) were associated with unsuccessful treatment outcomes. The JM showed a significant inverse association between weight gain and unsuccessful MDR-TB treatment outcomes. Therefore, weight gain may be used as a surrogate marker for good TB treatment response in Ethiopia.

## Introduction

Multidrug-resistant tuberculosis (MDR-TB) is a serious public health concern affecting approximately half a million individuals annually^1^ and is responsible for approximately one-third of all antimicrobial resistance (AMR) deaths globally^2^. Although the extent and burden of MDR-TB vary substantially, it is most severe in low-resource settings^3^. Globally, more than 90% of all MDR-TB cases originated in the 30 high tuberculosis (TB) burden countries^4^, with more than 50% being from Russia, India, and China^5^. In Ethiopia, the prevalence of MDR-TB cases was found to be 0.71% among new and 16% among re-treatment cases in 2018^5^. Data suggest that the key reasons for the continued emergence and spread of MDR-TB are inappropriate treatment of TB and person-to-person transmission^6,7^.

Treatment for MDR-TB cases is complex, arduous, and lengthy since it demands the use of second-line TB drugs, which are correlated with a higher risk of adverse drug reactions, poor outcomes, and higher costs^8^. Even though TB control has recorded good progress over recent decades, these gains are threatened by the current increase in the incidence of MDR-TB across many countries and a low treatment success rate globally at 59%^1,9^. Several factors can be associated with MDR-TB treatment outcomes. Comorbid conditions, including undernutrition^10,11^, Human Immunodeficiency Virus (HIV)^12,13^, and diabetes mellitus^14,15^, are among the factors associated with unsuccessful MDR-TB treatment outcomes. Undernutrition is recognised as a leading risk factor for TB incidence globally with a high population-attributable fraction for TB (20%), and can also affect TB treatment outcomes^5^. This impact has been exacerbated among patients with MDR-TB, with significantly prolonged sputum culture conversion and an increased likelihood of unsuccessful treatment outcomes^16,17^. Delayed nutritional recovery also impacts on the functional status and ability to return to work following patients’ cure^18^.

Nutritional interventions and counselling have been shown to be effective in improving body weight, total lean mass, and physical functioning^19^. The World Health Organization (WHO) recommends that all individuals with TB be assessed for their nutritional status and provided with adequate interventions^20^. There is also a great need to identify early indicators of a good response to treatment for patients with MDR-TB, as its treatment takes more than 18 months on average. The majority of patients with MDR-TB experience loss of appetite, which leads to significant weight loss^21^. Previous studies have provided evidence that a progressive increase in weight gain has been shown to have a substantial positive impact on TB outcomes^22–25^. Weight gain can therefore be a potentially important indicator of TB treatment responses.

In resource-constrained settings such as Ethiopia, the sputum culture test is resource-intensive, takes time, and is unavailable in most TB treatment centres. However, regularly measuring weight is a simple, low-cost, and practical tool to identify the most high-risk patients and enhance their quality of life^26^. Unfortunately, this practical clinical tool has received relatively little attention compared to its relevance in predicting treatment responses^23^.

Prior studies highlighted the relationship between body weight and TB treatment outcomes; however, these studies used classical statistical methods such as logistic regression or cox proportional hazards models^25–27^. These statistical approaches are unable to control for the time varying covariates^28^ thus producing spurious results^29^. Currently, a joint model (JM) is highly recommended to overcome such biased estimations in the presence of time-to-event and longitudinal data^30^. However, in Ethiopia, there is very limited evidence of longitudinal data analysis using JM to examine the effect of weight trajectories on unsuccessful MDR-TB treatment outcomes. Thus, this study aimed to determine the association between weight change during therapy and unsuccessful treatment outcomes among patients with MDR-TB in northwest Ethiopia.

## Methods

### Study design and settings

An institutional-based, multicentre, retrospective cohort study was carried out using de-identified data abstracted from the medical records of patients with MDR-TB commencing TB treatment from May 2015 to February 2022. The study areas included four selected Amhara public hospitals: The University of Gondar Comprehensive Specialised Hospital (UoGCSH), Debre Markos Comprehensive Specialised Hospital (DMCSH), Finote Selam General Hospital (FSGH), and Debre Tabor Specialized Hospital (DTSH). These hospitals were selected as they were in relatively politically stable areas and had high MDR-TB cases during the study period.

In Ethiopia, MDR-TB cases are diagnosed by rapid drug sensitivity testing (DST) using Xpert® MTB/RIF or line probe assay. People diagnosed with MDR-TB receive TB treatment in hospital settings free of charge. There are three treatment regimen options available based on the patient’s clinical and laboratory characteristics: (I) shorter regimen containing bedaquiline (Bdq), levofloxacin (Lfx) or moxifloxacin (Mfx), clofazimine (Cfz), pyrazinamide (Z), isoniazid high dose (HH), ethambutol (E), and ethionamide (Eto) for 9 to 12 months^31^, fully oral longer regimen containing Bdq-Lfx/mfx-Lzd-Cfz-cycloserine (Cs) for 18 to 20 months, and (III) individualized longer regimens containing at least 4 to 5 likely effective drugs with clinical expert decisions for 18–24 months.

In addition, the diagnosis of undernutrition among patients with MDR-TB is based on the Ethiopia Ministry of Health, which applies body mass index (BMI) as a screening tool. The treatment of undernutrition in patients with MDR-TB is tailored based on their nutritional status, defined into three care plans. Care plan A contains Ready to Use Therapeutic Foods (RUTF) and provided for patients with severe acute malnutrition (defined by a BMI less than 16). Care plan B contains Ready to Use Supplementary Foods (RUSF) or Plumpy nut and provided for patients with moderate acute malnutrition (i.e., BMI between 16.0 and < 17). Care plan C is designated for individuals with mild acute malnutrition (BMI between 17.0 and < 18.5) or those without acute malnutrition, which includes nutritional counselling services.

### Study population

The study population was all adults aged 15 and older living with drug-resistant tuberculosis (DR-TB) and attending TB treatment centres at the selected referral hospitals of the Amhara region from May 2015 to February 2022. We included patients having at least two records of body weight (one at baseline and one during follow-up) and treatment outcomes. Patients who were diagnosed with DR-TB but did not start TB treatment, transferred to other healthcare centres, and pregnant women were excluded from the analysis.

### Sample size and technique

We computed the minimum required sample size using the survival sample size determination formula as follows:

$$n=\frac{{\left({Z}_{\alpha /2}+{Z}_{\beta }\right)}^{2}}{{b}^{2}{p}_{1}{p}_{2}d}$$

where n = the required sample size, ${{\text{Z}}}_{\mathrm{\alpha }/2}$ = the critical value of the standard normal distributed at a 5% significance level, ${{\text{Z}}}_{\upbeta }$ = the critical value of the standard normal distributed at 20% of $\upbeta$, $\upbeta$ = type two error, b = ln (hazard ratio = 5.13)^32^, ${{\text{p}}}_{1}$= the proportion of patients with clinical complications (8.5%)^32^. ${{\text{p}}}_{2}$ = the proportion of patients without clinical complications (91.5%)^32^. d = the probability of event (10.8%)^32^.

After considering 15% missing values, the total sample size was found to be 430.

There were 526 MDR-TB medical records available between May 2015 and February 2022 at four TB treatment centres. Due to the limited number of cases, we then considered all available medical records during the study period.

### Data sources

Data were extracted from patients’ medical records using a checklist prepared in English, which was adapted from published articles and Ethiopian Federal Ministry of Health TB/MDR-TB treatment guidelines^17,21,33,34^. The necessary data were extracted from MDR-TB registration books, follow-up sheets, laboratory reports and medical records. The data abstraction tool included four major sections: (I) sociodemographic characteristics (residence, sex, age, marital status etc.)^31^, behavioural factors (smoking and alcohol drinking status), (III) TB treatment and clinical characteristics (Nutritional status, other comorbidities, previous treatment for TB, family history of MDR-TB, types of TB, drug side effects, serum electrolyte values etc.), and (IV) monthly follow-up sheet for weight, height, body mass index (BMI), culture, and Acid-fast bacillus (AFB).

The data abstraction tool was pretested for 10% of the total samples to check variable’s consistency and completeness of the data; necessary adjustments were made as needed before starting the actual data collection. Each data collector and supervisor had a master's degree in health science and sufficient knowledge about the disease. Moreover, two days’ orientation was provided to supervisors and data collectors on how and what information they should collect from the target data sources. The consistency of the extracted data was checked daily by the supervisor, and weekly feedback was provided by the principal investigator when deemed necessary.

### Missing data handling

We define a missing value as a specific important variable that was missing entirely or partially during the follow-up time. Eliminating observations or central statistics such as mean were considered for variables with few missing values. However, our data contained 22(5.3%) missing values for the variable of haemoglobin. Therefore, multiple imputations^2^ was performed to handle these missing values. Before applying MIs, Little’s test of missing completely at random (MCAR) test was considered to check whether the values were missing at random or not^35^. The final imputation was computed using a multivariate normal imputation model, including variables of residence, sex, occupation, nutritional and HIV status.

### Outcome measurements

The primary outcome of interest was unsuccessful treatment outcomes, which was defined as the composite of death, treatment failure, and loss to follow-up (LTFU). The main independent variable of the study was body weight change during treatment among patients with MDR-TB. The operational definition for the outcome and independent variables is available in the supplementary information (Table S1).

### Data management and descriptive analyses

Data were cleaned, coded, and entered into EpiData™ software Version-4.1 and transferred and validated in STATA/se version-17 and R version-4·2·0, where all statistical analyses were carried out. Descriptive statistics such as frequency, proportion, median, mean, and standard deviation were calculated to summarize the patient’s demographic and clinical characteristics. The Kaplan–Meier curves with the log-rank test were applied to compare the estimated event-free survival time for categorical variables and to estimate the median survival time of the study participants. Exploratory data analysis, such as individual profile plots of weight for 50 randomly selected participants, were constructed, and Q–Q plot was used to check the normality assumption.

### Statistical analyses

We performed three statistical models to address the research objectives.

Firstly, a linear mixed model with both random intercept and slope was fitted to identify predictors that were associated with weight trajectories (i.e., longitudinal sub-model); as we cannot ignore the longitudinal data measures that have variations within and between individuals.

Secondly, we fitted Cox proportional hazards regression models to identify predictors associated with outcomes in a time-to-event-based analysis (i.e., survival sub-model). Adjusted hazard ratio with 95% confidence interval (CI) and p-values were used to declare significant predictors of unsuccessful treatment outcomes.

Thirdly**,** we fitted a joint model (JM) considering the two models sharing some random effects, termed as “shared parameter model approach”^36,37^. This model was used to address the association between the weight variation over time and unsuccessful treatment outcomes.

**Figure 1:**
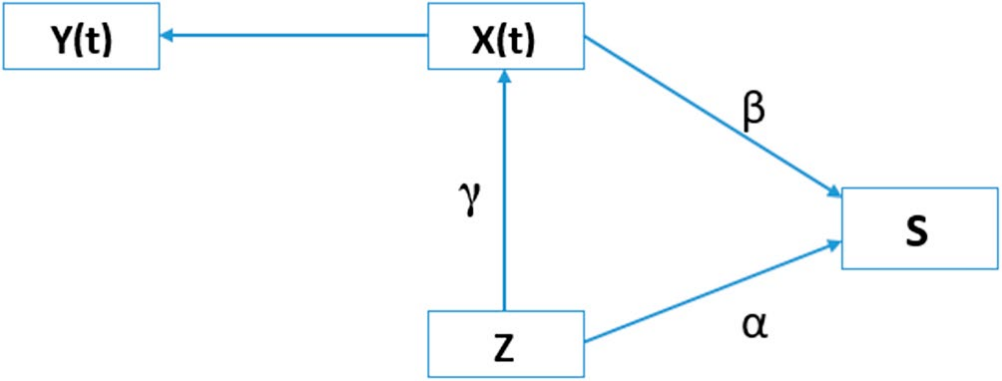
Causal diagram. Y(t), observed longitudinal data; X(t), weight trajectory function; S, survival; Z, main predictor (baseline underweight status); α, underweight effects on survival; γ, underweight effects on longitudinal process; β, effect of longitudinal process on survival.

The underlying causal diagram for joint modelling in this study is presented in Fig. 1.

For all models, we applied Akaike Information Criterion (AIC) value to select the best-fit model for the data. Those variables with a p-value < 0.25 in the bivariable analysis were eligible for the final multivariable analysis. Moreover, statistical significance was declared at p < 0.05, and all tests were two-sided.

### Ethics approval

Ethical approval was provided by the Australian National University Research Ethics Committee in 2022 (reference number: 2022/207) and the Amhara Regional Public Health Research Ethics Review Committee (reference number: NoH/R/T/T/D/5/22). All methods were performed in accordance with the organization’s guidelines and regulations. Participant’s verbal or written informed consent was not feasible because the study was based on existing MDR-TB medical records, and waiver of consent was granted. To ensure confidentiality of the information, personal identifiers such as name or cell phone number were not extracted from their medical charts.

### Consent for publication

All authors revised the manuscript and gave the consent to submit and publish the paper.

## Results

### Sociodemographic characteristics of the patients

Of the 526 patient medical charts available between May 2015 and February 2022, 107 records were excluded due to including ineligible study populations (children), or where records contained too many missing variables including weight measurements. Accordingly, a total of 419 patients with MDR-TB met the inclusion criterion and were analysed. The study participants had a mean age of 32.5 (SD ± 12.3) years, more than two-thirds (63.3%) were males, and 55.1% were from rural areas. One-third of (33%) participants were never married and unable to read and write. Table 1 presents the sociodemographic characteristics of the patients.

**Table 1:** Sociodemographic characteristics of patients with MDR-TB at the commencement of treatment at the Amhara referral hospitals of northwest Ethiopia between May 2015 and February 2022.

Variables	Frequency (N)	Percentage (%)
Residence		
Urban	188	44.9
Rural	231	55.1
Sex		
Male	265	63.3
Female	154	36.7
Age (years)		
15–24	108	25.8
25–34	196	46.8
35–45	55	13.1
> 45	60	14.3
Marital status		
Single	141	33.7
Married	244	58.2
Divorced	21	5.0
Widowed	13	3.1
Level of Education		
Cannot read and write	137	32.7
Primary	165	39.3
Secondary	79	18.9
Tertiary and above	38	9.1
Occupation		
Farmer	155	37.1
Employed	48	11.5
Unemployed	58	13.9
Labourer	44	10.5
Others	114	27.2
Religion		
Orthodox	383	91.4
Others*	36	8.6

Others* include Muslim and Protestant.

### Behavioural and clinical factors

The vast majority (91.8%) of the participants had no history of smoking and 87.6% had no history of alcohol drinking. Two hundred fifty-five (60.9%) participants were undernourished, and more than one-third (36.3%) were anaemic at initiation of TB treatment. Nearly one-quarter of the study participants (23%) had a family history of MDR-TB (Table 2).

**Table 2:** Behavioural and clinical factors among patients with MDR-TB receiving TB drugs at the Amhara referral hospitals of northwest Ethiopia between May 2015 and February 2022.

Variables	Frequency (N)	Percentage (%)
History of alcohol drinking		
Yes	52	12.4
No	367	87.6
History of smoking		
Yes	34	8.2
No	382	91.8
Baseline nutritional status (BMI)		
< 18.5 kg/m^2^	255	60.9
> 18.5 kg/m^2^	164	39.1
Baseline haemoglobin level		
Anaemic	152	36.3
Non-anaemic	267	63.7
Family history of MDR-TB		
Yes	97	23.1
No	322	76.9
MDR-TB Categories		
New	249	59.4
Re-treatment	170	40.6
History of prior injectable anti-TB medication		
Yes	137	33.7
No	282	66.3
Clinical complications during treatment		
Yes	139	33.2
No	280	66.8
Any existing comorbidity		
Yes	124	29.4
No	295	70.6
HIV status		
Positive	76	18.1
Negative	343	81.9
Diabetes mellitus		
Yes	9	2.1
No	410	97.9
Lung cavity on x-ray		
Yes	226	53.9
No/not done	193	46.1
Vitamin B6 supplement		
Yes	409	97.8
No	9	2.15

### Weight change and treatment outcomes

**Figure 2:**
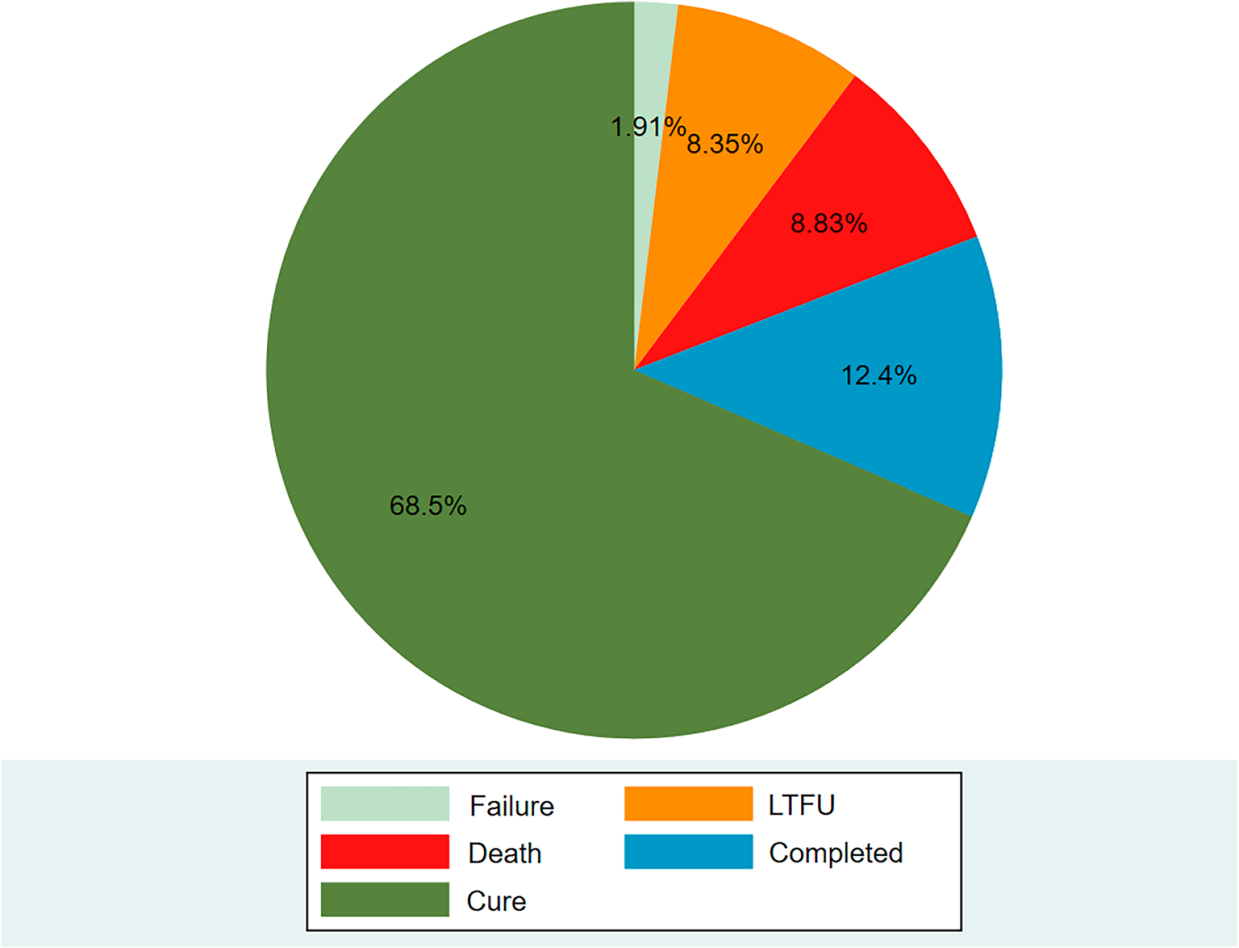
Treatment outcomes of patients with MDR-TB at the Amhara referral hospitals of northwest Ethiopia between May 2015 and February 2022.

After 24 months of follow-up, nearly one in five patients with MDR-TB had unsuccessful treatment outcomes, including 8.83% death, 8.35% LTFU, and 1.91% treatment failure (Fig. 2).

**Figure 3:**
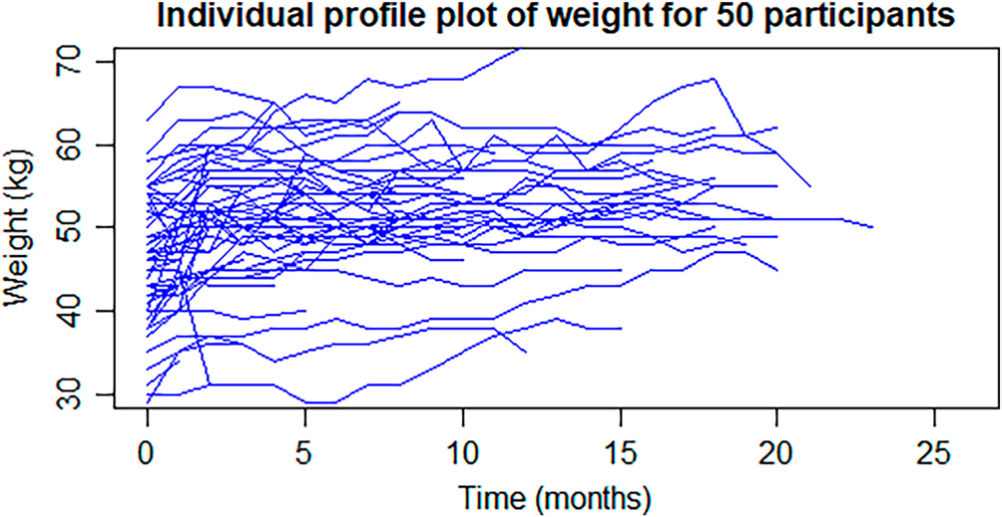
Individual profile plot of weight among the randomly selected 50 participants at the Amhara referral hospitals of northwest Ethiopia between May 2015 and February 2022.

Individual profile plots of 50 randomly selected participants described that weight varied significantly within individuals at TB treatment commencement and during follow-up time (Fig. 3). The mean weight of patients at baseline was 46.69(SD ± 8.30) kg and at the end of follow-up was 50.66 (SD ± 6.44) kg. In general, the mean weight of the participants increased by 0.32 kg per month in the first 12 months, while it increased by 0.18 kg in the second 12 months (Table 3).

**Table 3:** Average baseline and follow-up weight values for patients with MDR-TB at the Amhara referral hospitals of northwest Ethiopia between May 2015 and February 2022.

Follow-up period	Number	Weight in kg mean (± SD)
Baseline	419	46.69 (8.30)
3rd month	323	48.94 (8.10)
6th month	301	49.54 (8.02)
9th month	249	50.69 (7.93)
12th month	202	50.57 (8.11)
15th month	171	51.87 (7.77)
18th month	149	52.43 (7.47)
21st month	67	52.74 (7.28)
24th month	12	50.66 (6.44)

**Figure 4:**
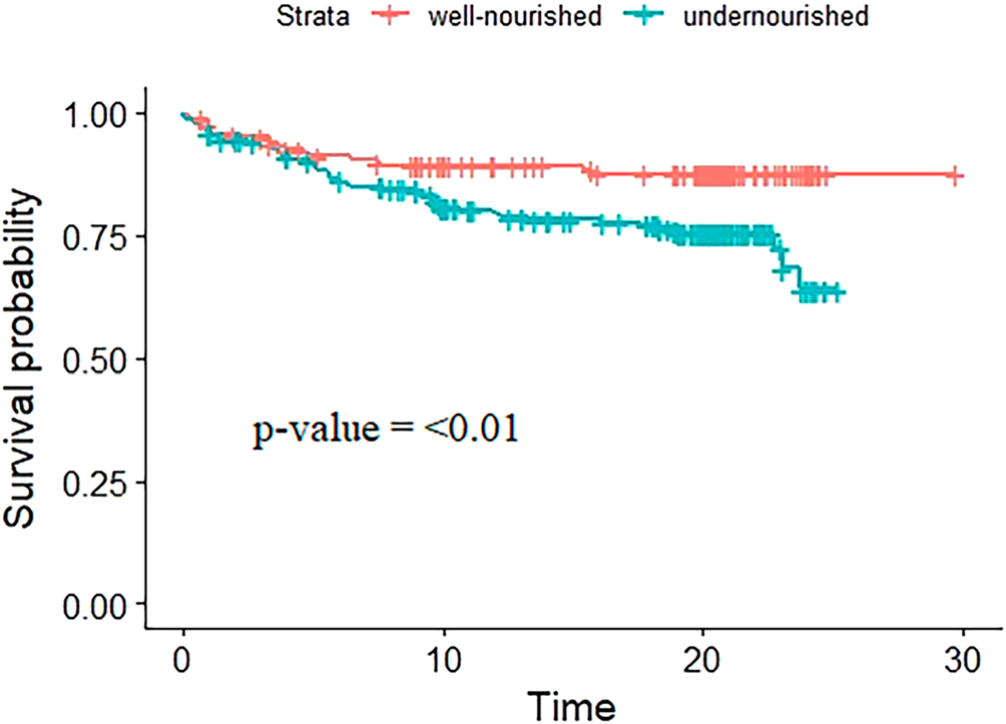
The Kaplan–Meier and log-rank survival estimates for time to unsuccessful treatment outcomes based on the nutritional status of patients with MDR-TB at the Amhara referral hospitals of northwest Ethiopia between May 2015 and February 2022.

In addition, there was a significant difference in survival across groups; the undernourished group had a lower survival time than the well-nourished group (Log-rank test p-value ≤ 0.01) (Fig. 4).

### Predictors of unsuccessful treatment outcomes

The potential predictors of unsuccessful treatment outcomes among adults with MDR-TB are shown in Table 4. After adjusting for confounders, undernutrition (AHR: 1.72, 95%CI: 1.10 to 2.97), HIV (AHR:1.79, 95%CI: 1.04 to 3.06), and clinical complications (AHR: 1.66, 95%CI: 1.03 to 2.67) were found to be significantly associated with unsuccessful treatment outcomes (Table 4).

**Table 4:** Event process joint model estimates to identify predictors of unsuccessful treatment outcomes among people living with MDR-TB at the Amhara referral hospitals of northwest Ethiopia between May 2015 and February 2022.

Variables	Event	Censored	AHR 95%CI	p-value
Residence				
Urban	42	146	1.53 (0.92, 2.54)	0.1
Rural	38	193	Ref	
Sex				
Male	51	214	1.44(0.78, 2.65)	0.23
Female	29	125	Ref	
Occupation				
Farmer	29	126	Ref	
Employed	7	41	0.53 (0.21, 1.33)	0.18
Unemployed	11	47	0.82 (0.35, 1.91)	0.64
Labourer	11	33	0.72 (0.31, 1.65)	0.44
Others	22	92	0.75 (0.40, 1.42)	0.38
History of Alcohol drinking				
Yes	8	44	Ref	
No	72	295	2.01 (0.91, 4.08)	0.06
Family history of MDR-TB				
Yes	17	80	1.13 (0.63, 2.01)	0.68
No	63	259	Ref	
History of prior injectable TB drug				
Yes	28	111	0.92 (0.57, 1.48)	0.78
No	52	228	Ref	
Nutritional status at baseline				
Undernourished	62	193	1.72 (1.10, 2.97)	0.04*
Well-nourished	18	146	Ref	
HIV status				
Positive	24	52	1.79 (1.04, 3.06)	0.03*
Negative	56	287	Ref	
Haemoglobin level				
Anaemic	41	111	1.51 (0.94, 2.44)	0.08
Non-anaemic	29	228	Ref	
Presence of clinical complications				
Yes	38	101	1.66 (1.03, 2.67)	0.03*
No	42	238	Ref	
Presence of lung cavity at baseline				
Yes	45	181	0.87 (0.54, 1.38)	0.55
No	35	158	Ref	
Association			0.96(0.94, 0.98)	< 0.001*

*Statistically significant predictors at p-value <0.05.

The JM also revealed a significant association between weight variation over time and early unsuccessful outcomes, with a one-unit increase in weight corresponding to a nearly 5% reduction in unsuccessful outcome risk (AHR: 0.96, 95% CI: 0.94 to 0.98) (Table 4).

## Discussion

In this multicentre retrospective cohort study, we sought to determine the association structure between weight trajectories and MDR-TB treatment outcomes. One in five (19%) MDR-TB cases had unsuccessful treatment outcomes, including 8.83% deaths, 8.35% loss to follow-up, and 1.91% treatment failures. Our study showed a significant association between longitudinal weight variation and time to unsuccessful treatment outcomes. The survival analysis also identified that living with HIV, undernourishment, and the presence of clinical complications such as pneumothorax were significant predictors of unsuccessful treatment outcomes.

The composite unsuccessful treatment outcome proportion in our cohort study was 19%, which is consistent with studies conducted in other Ethiopian settings^34,38^, Niger^10^, and China^39^. However, our finding is lower than what was reported in other countries^40–43^. Another study conducted in Sudan showed that 36.5% of patients with MDR-TB had unsuccessful treatment outcomes^44^. A systematic review and meta-analysis also reported that the pooled percentage of unsuccessful treatment outcomes was 35.4%^45^. These observed variations might be due to multifaceted factors. One possible contributing factor is introduction of a short-term drug regimen and advancing care provision over time. For instance, bedaquiline and linezolid are now the most potent drugs (i.e., good bactericidal and sterilizing profiles) that may improve the MDR-TB treatment success rates and support achieving the WHO targets^46^. In addition, the low treatment success rates in other settings might be due to the high percentages of patients’ loss to follow-up, which has been globally identified as a significant challenge to treating MDR-TB successfully^47^. Provided that previous studies have reported > 29% loss to follow-up rates^40,42,43^. Other possible reasons may include differences in study areas, sample sizes, follow-up periods and sociodemographic characteristics. Our findings therefore suggest that simplifying complex treatment regimens, implementing adherence strategies, and providing some economic support may be paramount to the success of treatment programmes.

Body weight change during treatment was found to be associated with a significant decrease in the hazard of unsuccessful treatment outcomes, with a one-unit increase in weight corresponding to a nearly 5% reduction in unsuccessful treatment outcome risk. This finding is consistent with studies conducted in Philippines and Vietnam showed that a 5% weight gain from baseline weight during treatment was correlated to a lower risk of poor outcomes^48,49^. Other studies also indicated that the patient with inadequate weight gain during treatment were significantly more likely to have unsuccessful treatment outcomes^27,49,50^. This finding indicates that weight gain over time may serve as an important biomarker for successful treatment outcomes and how patients progress during TB treatment.

In survival analysis, baseline undernutrition has also been found to be an independent predictor of unsuccessful treatment outcomes, consistent with findings of prior studies^16,31,51^. The WHO guideline reports^20^ indicated that patients with undernutrition and failure to gain weight during follow-up are more likely to have adverse drug reactions and poor outcomes. Our recent meta-analysis also found that baseline undernutrition was associated with prolonged time to sputum culture conversion and unsuccessful treatment outcomes in adults with MDR-TB^16^. This is due to the fact that undernutrition significantly impairs cell-mediated immunity, cytokine production, phagocytic function, and antibody concentration^52,53^. Undernutrition also reduces gastrointestinal drug absorption, which may lead to sub-therapeutic or supra-therapeutic drug levels and adverse drug reactions^23,54,55^. This implies the necessity of extra care and scaled-up supports like financial and food basket supports for undernourished patients from the beginning of TB treatment (i.e., “hit hard, hit early” principle). Currently, all undernourished TB patients are eligible to receive nutritional therapy such as Plumpy-Nut or Ready to Use Therapeutic Foods (RUTF)^21^. Such interventions would have the potential to substantially improve nutritional recovery and weight gain during follow-up time^56^. Therefore, strengthening nutritional interventions and close monitoring of undernourished patients can further improve their weight gain and subsequent treatment outcomes.

Consistent with other studies^13,17,38^, we found that the likelihood of unsuccessful treatment outcomes was highest in people with MDR-TB and HIV coinfections. A systematic review and meta-analysis in sub-Saharan Africa also reported that the risk of unsuccessful treatment outcomes was higher in people with MDR-TB and HIV coinfections than in their counterparts^57^. This might be explained by the synergetic effects of the two diseases; this population is more likely confronted by overlapping or additive adverse drug reactions owing to high pill burdens, drug-drug interactions, and poor treatment adherence^58,59^. In addition, diagnosing MDR-TB in people living with HIV is more challenging and often misdiagnosed with other pulmonary infections, delaying the initiation of treatment^58^. The degree of low immune status in this population may also be high which leads to an increased chance of other opportunistic infections. However, the treatment success rate can be improved by scaling up integrated TB-HIV services.

Furthermore, those with clinical complication such as pneumothorax has been found to associate with a higher risk of unsuccessful treatment outcomes^32,60^. This may indicate the severity of the disease, the occurrence of adverse drug reactions, and impaired immunity, which increases the risks of life-threatening conditions.

Collectively, our findings highlight the significance of weight gain and TB comorbidities as core tenets to improve treatment outcomes in patients with MDR-TB. Our findings would also be relevant to support the design and implementation of future integrated and targeted interventions to treat the most clinically vulnerable. The interventions could include integrated HIV-TB services, adverse event monitoring, and nutritional support in an effort to improve the MDR-TB treatment success rate in Ethiopia. Importantly, undernutrition has been recognised as playing an inextricable role in TB treatment that needs special attention to tackle its consequences. In Ethiopia, programmatic interventions such as scaling up actions on addressing undernutrition must be accelerated and expanded. In particular, regularly monitoring patient’s nutritional status and providing appropriate nutritional intervention can increase weight gain and subsequently improve nutritional recoveries as well as MDR-TB treatment outcomes.

### Limitations of the study

Our study has some important limitations that need to be kept in mind when interpreting the findings. Due to its retrospective nature, some important variables, such as socioeconomic and nutritional-related factors (food security and dietary diversity), were not considered in this study. In addition, the weight trajectory function between the MDR-TB treatment regimens (short versus to long) could not be demonstrated due to lack of adequate data. The medical records also had incomplete information regarding the length of time between diagnosis and treatment initiation, treatment adherence, and liver and renal function tests. Such data would be essential for future analyses to examine the impact of treatment delays or organ dysfunction on MDR-TB treatment outcomes.

Furthermore, we solely included four TB treatment sites which might not be applicable to other countries. Therefore, we recommend that well-powered prospective longitudinal studies should be performed to confirm our findings and fill the gaps.

## Conclusion

We found a strong association between body weight change and treatment outcomes in which patients with body weight gain showed a successful treatment outcome. Moreover, HIV coinfection, undernutrition, and clinical complications such as pneumothorax were identified as significant predictors of unsuccessful treatment outcomes. Therefore, those patients with weight loss in early TB treatment should be targeted as part of routine clinical evaluation to make appropriate decisions. Nutritional interventions and supports should be prioritized to tackle the burden of undernutrition effectively. In addition, closer follow-up and provision of social protection services should be strengthened. Further research using a multicentre prospective cohort study is needed to evaluate the long-term consequences of weight trajectories on chronic lung sequelae and treatment relapse.

### Supplementary Information

Supplementary Table S1.

## Data Availability

The datasets used in the study are available from the corresponding author on reasonable request.
