# Supplementary material for: Impacts of body weight change on treatment outcomes in patients with multidrug-resistant tuberculosis in Northwest Ethiopia
Source: Sci Rep. 2024 Jan 4;14:508. doi: 10.1038/s41598-023-51026-y (PMC10767082; doi:10.1038/s41598-023-51026-y)
Supplement: Supplementary file 1 — Supplementary Table S1. [file 41598_2023_51026_MOESM1_ESM.docx]

**Table S1: Operational definitions**

| **Terms** | **Definitions** |
| --- | --- |
| **Cured** | When patients completed the treatment regimen, and three consecutive negative cultures were collected separately at least 30 days after the intensive phase.[^1^](#_ENREF_1) |
| **Treatment completed** | Patients who completed the treatment regimen lacked three consecutive negative cultures and showed no evidence of failure.[^1^](#_ENREF_1) |
| **Treatment failure** | When treatment was discontinued, or regimen was changed by at least two drugs due to adverse reactions, culture remained positive at the end of the intensive phase, or culture reverted in the continuation phase after conversion to negative, or acquired resistance to fluoroquinolones, or second-line injections was confirmed.[^1^](#_ENREF_1) |
| **Loss to follow-up** | When treatment was interrupted for more than 2 months.[^1^](#_ENREF_1) |
| **Death** | Death was defined as death from any cause during the course of treatment.[^1^](#_ENREF_1) |
| **Transferred out** | Patients who were transferred to another TB treatment centre during treatment.[^1^](#_ENREF_1) |
| **Successful treatment outcomes** | A composite of cured and treatment completed.[^1^](#_ENREF_1) |
| **Unsuccessful treatment outcomes** | A composite of treatment failure, loss to follow-up, and death.[^1^](#_ENREF_1) |
| **MDR-TB** | It is resistant to at least the two potent first-line TB drugs (i.e., rifampicin and isoniazid). |
| **Undernutrition** | Individuals who have low BMI values (<18.5 kg/m2).[^2^](#_ENREF_2) |
| **Anaemia**: | Serum haemoglobin level < 12g/dl for females and 13g/dl for males.[^3^](#_ENREF_3) |

**References:**

1. Organization WH. *Definitions and reporting framework for tuberculosis–2013 revision: updated December 2014 and January 2020*. Report no. 9241505346, 2013. World Health Organization.

2. CDC. Healthy Weight, Nutrition, and Physical Activity; About Adult BMI. June 3, 2022.

3. Organization WH. *Haemoglobin concentrations for the diagnosis of anaemia and assessment of severity*. 2011. World Health Organization.
